# All-Inorganic Lead-Free Doped-Metal Halides for Bright Solid-State Emission from Primary Colors to White Light

**DOI:** 10.1021/acsami.3c06546

**Published:** 2023-07-17

**Authors:** Ramavath Babu, Iago López-Fernández, Seelam Prasanthkumar, Lakshminarayana Polavarapu

**Affiliations:** † School of Chemistry, University of Hyderabad, Gachibowli, Hyderabad 500 046, India; ‡ Polymer and Functional Materials Division, CSIR-Indian Institute of Chemical Technology (IICT), Tarnaka, Uppal Road, Hyderabad 500 007, India; § Academy of Scientific and Industrial Research (AcSIR), Ghaziabad, Uttar Pradesh 201 002, India; ∥ CINBIO, Materials Chemistry and Physics Group, University of Vigo, Campus Universitario Marcosende, Vigo 36310, Spain

**Keywords:** metal halides, doping/alloying, solid-state emitters, luminescence, self-trapped emission

## Abstract

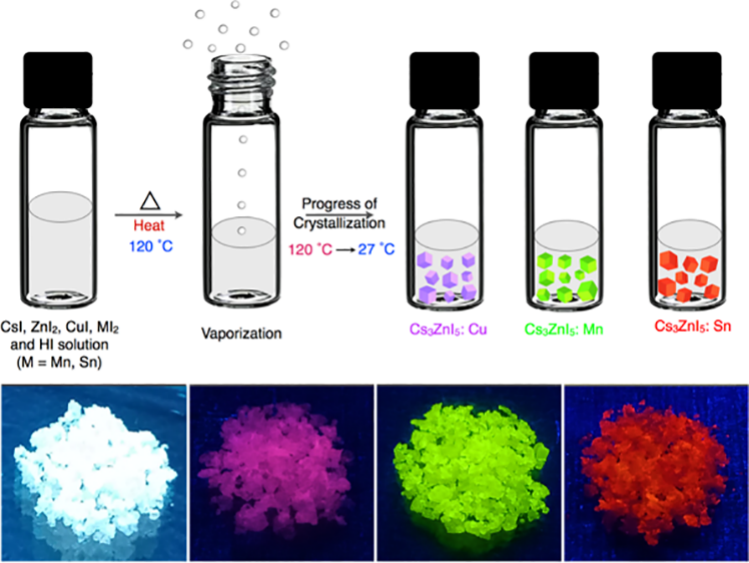

Metal halides have been explored with the aid of strong photoluminescence for optical and optoelectronic applications. However, the preparation of lead (Pb)-free solid-state emitters with high photoluminescence quantum yields (PLQYs) and tunable emission remains exceptionally challenging. Herein, we report metal ion (Cu(I), Mn(II), and Sn(II))-doped Cs_3_ZnI_5_ single crystals that are primary color (violet, green, and orange/red) emitters with extremely high PLQYs. Whereas the Mn-doping leads to bright green emissions with 100% PLQY, the Cu- and Sn-doping give rise to blue and red emissions with PLQYs of 57 and 64%, respectively. Interestingly, higher Mn doping results in white light emissive crystals as a side product, which are found to be Mn-doped CsI single crystals. The bright white light emissive crystals can be synthesized in a pure form in large quantities and exhibit a high color rendering index (CRI) of 78 and CIE coordinates of (0.30, 0.38), which are close to daylight conditions. To the best of our knowledge, this is the first demonstration of white light emission from a complete inorganic system. Importantly, the single crystals of all colors exhibit high long-term stability as their PLQY remains unchanged even after 2 months of preparation, and are thermally stable up to 600 °C.

## Introduction

Solid-state light-emitting materials have played an important role in the development of modern science and technology, and they have a wide range of applications including communication, data storage, lightning, flat panel display, photonics, and optoelectronics.^1−8^ The generation of primary colors (blue, green, and red) as well as white light is critical for lighting and display devices.^9−16^ The commercially successful solid-state lighting (SSL) devices typically consist of light-emitting diodes (LEDs) coated with a single or mixture of phosphors, which are based on transition-metal or rare-earth ion-doped high-bandgap host materials, for example, a blue LED coated with a yellow phosphor (YAG:Ce^3+^) or an ultraviolet LED (InGaN–AlGaN) coated with red, green, and blue phosphors.^17,18^ On the other hand, with the emergence of colloidal semiconductor nanocrystals (NCs) such as core–shell-type cadmium chalcogenides,^19,20^ indium phosphides,^21,22^ and lead halide perovskites^23−28^ with high photoluminescence quantum yields (PLQYs) have been exploited as primary colors for display applications. However, their progress in terms of commercial usage is limited by the issues associated with either toxicity or long-term stability. In addition, it is challenging to retain the PLQY in a solid state as high as in a colloidal solution.^29^ This is because the solution processing of colloidal NCs into thin films requires a colloidal solution that is free of ligands, which requires a purification process. However, this can lead to the detachment of surface ligands, leading to defects and thus resulting in a decrease of PLQY.^30^

Recently, low-dimensional metal halides with a wide band gap have emerged as promising candidates for SSL and display technologies as they exhibit long lifetimes, low power consumption, and high efficiency when compared to traditional incandescent and fluorescent lighting sources.^31−39^ Recent studies demonstrate that these wide-band gap metal halides generally emit PL upon doping with metal ions or through the introduction of organic molecules in their lattice.^36,40−45^ The emission from these materials has been attributed to electronic transitions of dopants or self-trapped states.^32,36,40−44^ Despite different host metal halides and different dopants reported in the literature, the generation of all primary colors with a single host matrix has not yet been reported. On the other hand, the layered metal halides reported for white light emission are mainly Pb-based organic–inorganic hybrid systems.^35−37^ However, besides the toxic Pb, the organic component of hybrid systems can make them intolerant to high temperatures and long-term stability. Currently, the development of all-inorganic, Pb-free, and solid-state emitters with tunable emission (primary colors and white light), high PLQY, and long-term stability remains an outstanding challenge in the field of metal halide emitters.

**Figure 1:**
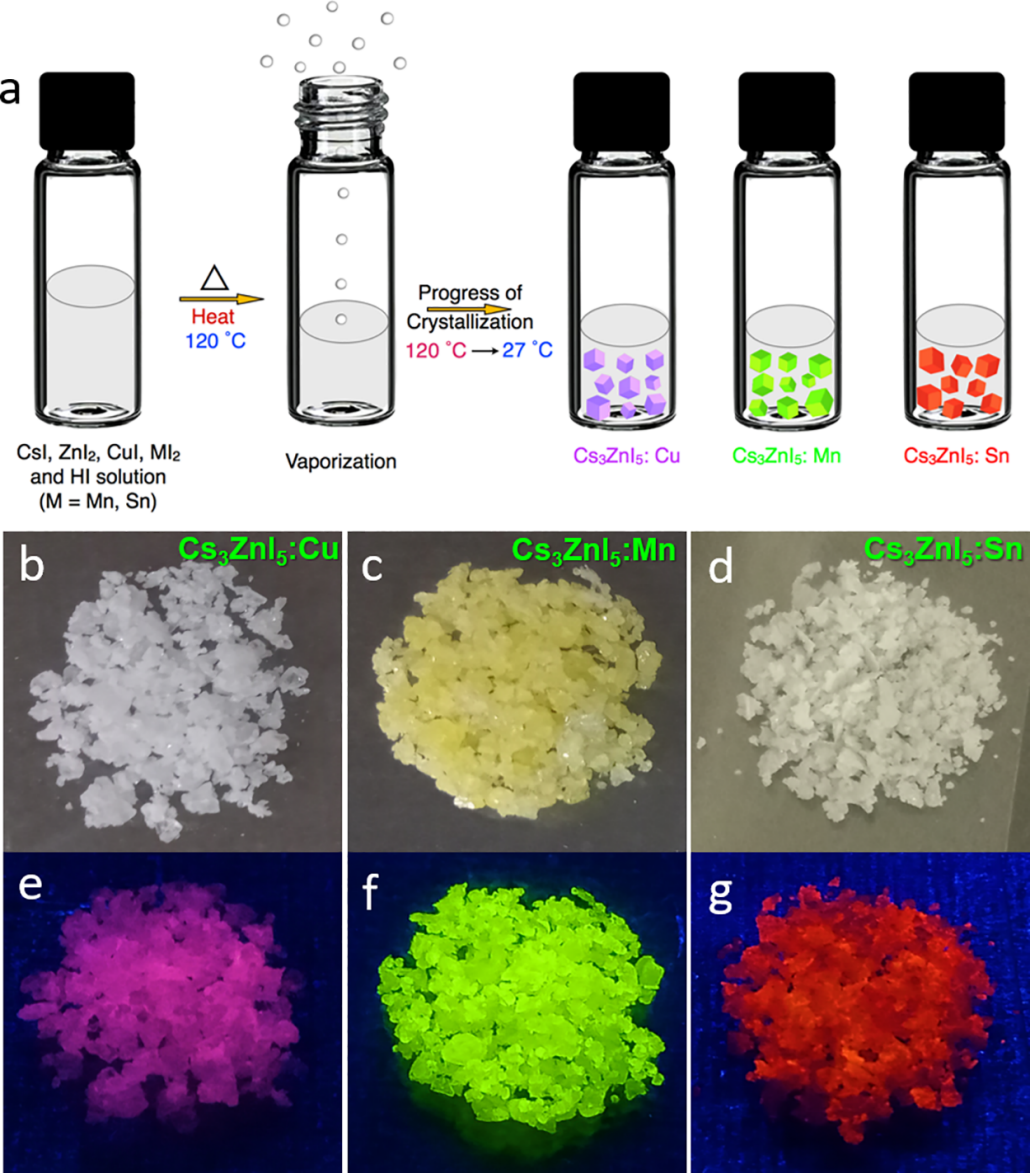
(a) Schematic illustration of the preparation of single crystals of Cs_3_ZnI_5_:M (where M = Cu, Mn, and Sn). The photographs of the as-prepared single crystals under daylight (b–d) and UV light (e–g).

In this work, we present all-inorganic, Pb-free, solid-state light emitters based on metal ion-doped wide-bandgap Cs_3_ZnI_5_, and CsI metal halides. While the doping of Cu(I), Mn(II), and Sn(II) into Cs_3_ZnI_5_ leads to violet, green, and orange-red emissions with 57%, 100%, and 64% PLQY, respectively, the doping of Mn(II) into a CsI matrix results in an intense white light emission (Figure 1). Whereas the green emission from Mn(II)-doped Cs_3_ZnI_5_ is attributed to electronic transitions in tetrahedral coordinated Mn(II) ions, the other colors are assigned to emission from self-trapped states. It demonstrates the tunability of self-trapped states by the dopant metal ions. The CRI of the white light emission from Mn(II)-doped CsI crystals is ∼78, which is close to the most commercially available LEDs. Despite several reports on white light emission from organic–inorganic hybrid metal halides, this is the first report on an all-inorganic metal halide system. Long-term stability measurements and TGA analysis prove that the PLQYs of doped materials are stable for more than 2 months and thermally sustain without decomposition at high temperatures (>600 °C).

## Results and Discussion

### Synthesis and Structural Studies of Cs_3_ZnI_5_:M (M = Cu^+^, Mn^2+^, and Sn^2+^) Crystals

**Figure 2:**
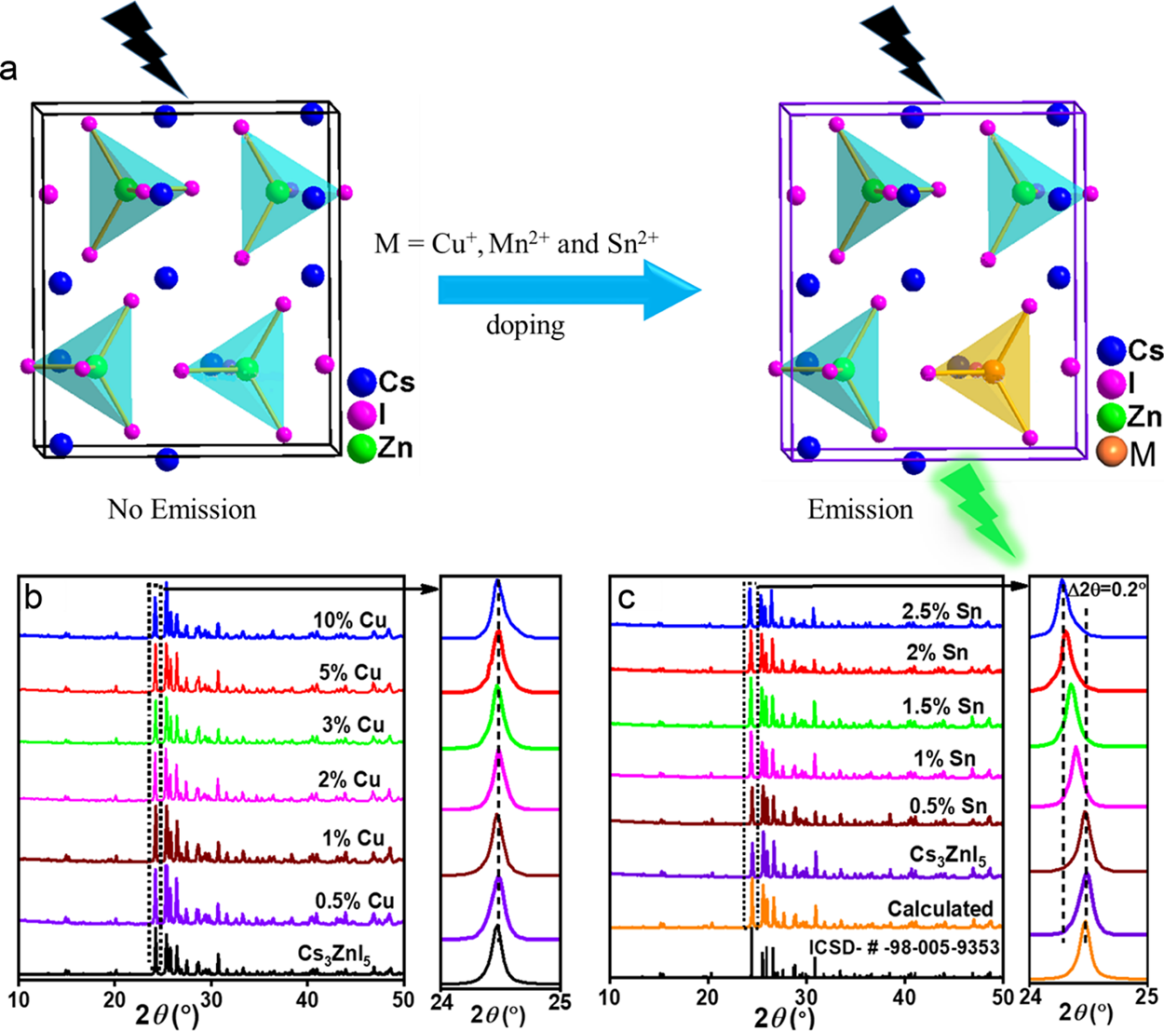
(a) Unit cell of the crystal structures for pristine and Cu, Mn, and Sn-doped Cs_3_ZnI_5_ obtained from the refinement of single-crystal X-ray diffraction data (b, c) powder XRD diffraction patterns for Cu and Sn-doped Cs_3_ZnI_5_ with different dopant concentrations.

The large single crystals of Cs_3_ZnI_5_ and Cs_3_ZnI_5_:M (M = Cu^+^, Mn^2+^, and Sn^2+^) were prepared through the temperature-lowering crystallization (TLC) method by dissolving the corresponding precursors (ZnI_2_, CsI, CuI, MnI_2_, and SnI_2_) in HI solution (Figures 1 and 2a, Schemes S1 and S2, and see the Experimental Section in the Supporting Information for more details).^46^ As the precursors started to crystallize in sample vials, the doped-Cs_3_ZnI_5_ single crystals emit strong PL under UV light illumination (Figure S1), while the undoped crystals are nonemissive. As shown in Figure 1, the sizes of the crystals are in the range of a few micrometers to millimeters, and they emit strong PL under UV light. The crystals emit violet, green, and orange-red PL upon doping with Cu(I), Mn(II), and Sn(II), respectively (Figure 1). The large single-crystalline nature of the samples has enabled their structural characterization by single-crystal XRD (see the Supporting Information for details). The X-ray data of pristine Cs_3_ZnI_5_ crystallizes show that four iodide ions are connected to the Zn^2+^ ion tetrahedral ZnI_4_^2–^ unit, which is surrounded by I^–^ and Cs^+^ ions. The lattice parameters obtained by the refinement of the Cs_3_ZnI_5_ X-ray data are in good agreement with the literature (Table S1).^47^ The incorporation of Sn (Cs_3_ZnI_5_:Sn) results in a slight increase in the lengths of unit cells compared to the pristine sample (Table S1). Furthermore, the single-crystal XRD data collected from randomly selected crystals of Cs_3_ZnI_5_:Sn with different amounts of Sn-dopants (1.5, 2, and 2.5%) revealed that the lattice parameters are gradually increased with Sn-concentration (Tables S2 and S3). Similarly, the variation of bond distances and bond angles is also observed for Cs_3_ZnI_5_:Sn crystals in comparison to that of undoped crystals (Tables S4 and S5). The increase in lattice constants upon Sn-doping is consistent with a larger size of the Sn^2+^ ion (1.0 Å) compared to the Zn^2+^ (0.60 Å) ion.^47^ However, Cu- and Mn-doping results have not shown differences in lattice parameters due to small differences in the sizes of Zn^2+^ (0.60 Å), Cu^2+^ (0.60 Å), and Mn^2+^ (0.66 Å) ions.

Furthermore, the phase purities of the pristine and doped (Cu, Mn, and Sn)-Cs_3_ZnI_5_ samples were investigated by powder X-ray diffraction (PXRD) measurements (Figures 2b,c). The diffraction patterns of Cs_3_ZnI_5_ closely resemble the calculated and ICSD (#98-005-9353) patterns, indicating that the prepared crystals are pure and have only one crystalline phase (Figure 2b and Figure S2). Moreover, the crystalline phase of doped crystals retains the isomorphic structure of the pristine sample as their diffraction patterns are undistinguishable (Figure 2b and Figure S2). However, for Sn-doped samples, the diffraction peak shifts toward lower angles with increasing Sn concentration (Figure 2c). This is because of the expansion of the lattice by the replacement of Zn^2+^ with a larger size Sn^2+^, and it is in accordance with the results of the single-crystal XRD data (Table S2). On the other hand, no shift in diffraction peaks is observed for Cs_3_ZnI_5_:Cu and Cs_3_ZnI_5_:Mn samples due to less variation in the sizes of Cu^+^ and Mn^2+^, suggesting that the lattice neither expands nor contracts upon doping these ions (Figure 2b and Figure S2).

The dopant concentrations were controlled by varying the amounts of dopants in the precursors. The exact dopant (Cu, Mn, and Sn) concentrations incorporated in the single crystals were investigated by inductively coupled plasma optical emission spectroscopy (ICP-OES) measurements (Tables S6a–d). The concentrations of Cu and Mn dopants obtained from ICP-OES analysis are almost similar to precursor concentrations, suggesting that the doping is very efficient. However, in the case of Sn, it matches well with the precursor concentrations at low dopant concentrations (up to 1.5%), and then gradually decreased from precursor concentrations (2 and 2.5% Sn-dopant). These results suggest that Sn^2+^ does not replace Zn^2+^ as well as Cu and Mn because of its large size, and as well the +2 oxidation state of Sn is unstable, and it tends to transform into +4. In addition, the elemental compositions of the doped-single crystals were measured by energy-dispersive X-ray spectroscopy (EDS) coupled with scanning electron microscopy (SEM) (see Table S6b–d for detailed analysis). The elemental mapping of different dopants shows their homogeneous distribution in the Cs_3_ZnI_5_ crystal lattice (Figure S3). However, at high concentrations of Cu (>4%), Mn (>40%), and Sn (>2.5%), some impurity crystals with different compositions were observed in PXRD analysis. Whereas the Cu-doped Cs_3_ZnI_5_ has CsCu_2_I_3_ impurity, the Mn and Sn-doped Cs_3_ZnI_5_ samples have white CsI:Mn single crystals and black crystals of Cs_2_SnI_6_, respectively, as impurities (Figures S4 and S5). It is worth mentioning that the oxidation states of Cu(I) and Mn(II) ions remain unaltered even in impurities; however, both II and IV oxidation states were observed for Sn. Further, the oxidation states of Cu^+^ and Mn^2+^ are confirmed by EPR spectroscopy (Figure S6). The Cu^+^ ion has a d^10^ electronic configuration that is not EPR active, while the Mn(II) has a d^5^ electronic configuration giving a very characteristic EPR spectrum (Figure S6). The valance state of Sn is studied by employing X-ray photoelectron spectroscopy (XPS) (see Figure S7 for detailed analysis). The typical Sn 3d_5/2_ and 3d_3/2_ peaks appear at 486.8 and 495.9 eV, respectively, confirming the presence of Sn^2+^ ions in the crystal.^48,49^ However, it should be noted that XPS analysis cannot differentiate Sn^2+^ and Sn^4+^ oxidation states.^49^ Nevertheless, it is known that Sn^2+^ ion incorporation results in STE emission with a large Stokes shift while Sn^4+^ is not emissive.^49^

### Optical Properties of Cs_3_ZnI_5_:M (M = Cu^+^, Mn^2+^, and Sn^2+^) Crystals

**Figure 3:**
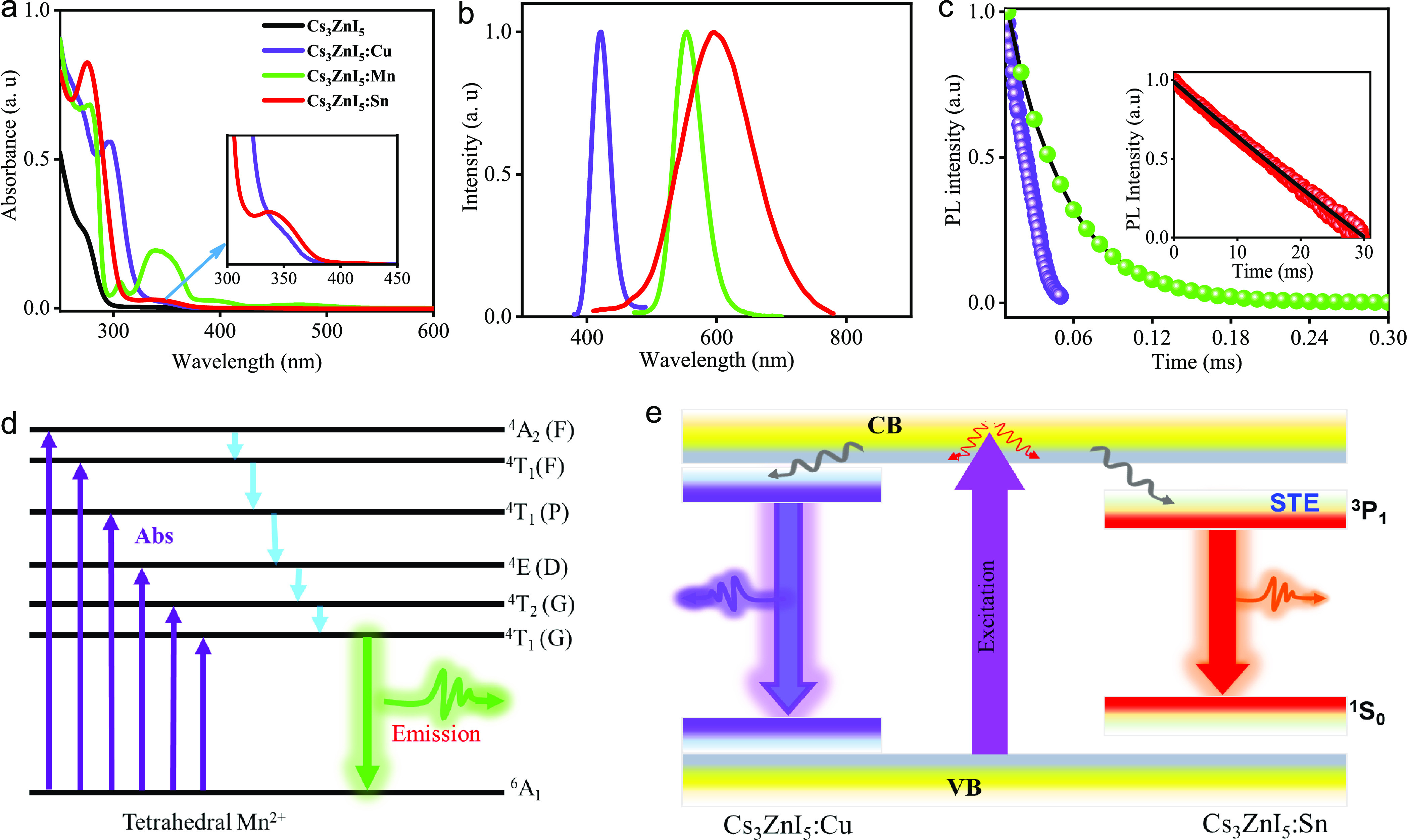
Absorption (a), photoluminescence (b), and time-resolved PL lifetime (c) data of C_3_ZnI_5_:M (where M = Cu, Mn, and Sn) single crystals. The absorption spectra are obtained from the diffuse reflectance spectral (DRS) measurements by applying Kubelka–Munk function. (d) Schematic illustration for the origin of PL emission in Mn-doped Cs_3_ZnI_5_, and (e) Cu and Sn-doped C_3_ZnI_5_ metal halide systems.

Figure 3a shows the absorption spectra of the pristine and M (M = Cu^+^, Mn^2+^, and Sn^2+^)-doped Cs_3_ZnI_5_ crystals. The spectra are obtained from the diffuse reflectance spectra (DRS) measurements by applying Kubelka–Munk function. The pristine Cs_3_ZnI_5_ shows a broad absorption with a peak maximum at 280 nm. Whereas the Cs_3_ZnI_5_:Cu and Cs_3_ZnI_5_:Sn samples show a new broad absorption peak at 346 nm due to the formation of intra-gap states upon doping, the Cs_3_ZnI_5_:Mn sample exhibits multiple absorption peaks at 298, 314, 382, 394, 469, and 486 nm, which are corresponding to ^6^A_1_ → ^4^A_2_ (F), ^6^A_1_ → ^4^T_1_ (F), ^6^A_1_ → ^4^T_1_ (P), ^6^A_1_ → ^4^E (D), ^6^A_1_ → ^4^T_2_ (G), and ^6^A_1_ → ^4^T_1_ (G) d-d transitions in Mn^2+^ tetrahedra (Figures 3a,d).^50−52^ While the bandgap derived from the Tauc plot of the absorption spectra of Cs_3_ZnI_5_ is 4.33 eV, the intra-gap energy states after doping with Cu and Sn are 3.43 and 3.35 eV, respectively (Figure S8). It should be noted that the absorption band of Cs_3_ZnI_5_ at 280 nm remains unaltered after doping with Mn and Sn, but Cu doping leads to a slight red shift with a peak at 295 nm. These results suggest that Cu^+^-doping affects the electronic absorption spectra of the host Cs_3_ZnI_5_ (Figure 3a, see Figure S9 for PLE spectra).

As the prepared samples emit strong photoluminescence and dopant-dependent emission color under UV light excitation, PL properties of the solid samples (pristine and Cu^+^, Mn^2+^, and Sn^2+^-doped Cs_3_ZnI_5_) were investigated at room temperature (RT) and are depicted in Figure 3b (also, see Figure S10 for dopant concentration-dependent emission spectra). Although the pristine sample is nonemissive, the Cu^+^-, Mn^2+^-, and Sn^2+^-doping leads to violet, green, and orange-red emission with peak maxima at 422, 555, and 596 nm and with full-width at half maximum (FWHM) results of 30, 51, and 129 nm, respectively (Figure 3b). These results demonstrate the fabrication of inorganic, Pb-free, solid-state primary color emitters by varying the type of dopant in a wide-bandgap 0D host matrix. Subsequently, the effect of dopant concentration on the PL properties of the prepared crystals is systematically studied. While there is no change in the shape of the PL spectra, a gradual increase in intensity was observed with increasing the concentration of Cu, Mn, and Sn ions from 0.5 to 3%, 1 to 40%, and 0.5 to 1.5%, respectively (Figure S10). However, the PL intensity starts decreasing with further increasing the dopant concentration due to the concentration quenching effect (Figure S10 and Table S7). The highest PLQYs obtained for Cu-, Mn-, and Sn-doped crystals are 57, ∼100, and 64% with dopant concentrations of 3, 40, and 1.5%, respectively. Time-resolved PL measurements revealed the inverse correlation between the emission energy and decay lifetime. The Cu, Mn, and Sn-doped samples exhibit a monoexponential PL decay with lifetimes of 23 μs, 44 μs, and 9.8 ms, respectively (Figure 3c, see Table S7 for a summary of PLQs and PL lifetimes). These results are extremely promising that it is possible to obtain Pb-free solid-state primary color emitters simply by changing the type of dopant of the 0D host matrix. The color tunability and high PLQY of the samples in the solid state could be very promising for the replacement of Mn^2+^- or Pb^2+^-based organic–inorganic hybrid systems for light-emitting applications.^50−53^

Generally, the origin of dopant-induced emission in metal halides is attributed to (1) the formation of intra-gap electronic states that leads to self-trapped emission (STE),^35^ (2) the energy/electron transfer from the host to the dopants,^54^ and (3) the formation of new emissive complexes between the dopants and halides of the host.^40,41^ The long PL lifetimes also suggest that the transitions are either forbidden or self-trapped emission. The shape and position of the PL spectra are independent of the excitation wavelength for all the samples (Figure S11), suggesting the presence of only single emissive species in all the samples.^55,56^ Here, in the case of Mn-doping, the absorption spectra and PXRD studies clearly indicate the presence of tetrahedral coordinated Mn^2+^ ions in the host matrix.^44^ It has been well studied that Mn^2+^ tetrahalide complexes emit green PL with a peak maximum around 500–550 nm depending on the coordination geometry of the Mn^2+^ ions, the strength of the crystal field, and the ligand environment, while an octahedral Mn^2+^ leads to an orange–red emission.^52,53^ Therefore, it is likely that the narrow green emission from Cs_3_ZnI_5_:Mn originates from the ^4^T_1_ → ^6^A_1_ transition in Mn^2+^ tetrahedral, as illustrated in Figure 3d. On the other hand, the Cu^+^ and Sn^2+^ tetrahalide complexes likely induce intra-gap states that lead to trapped exciton emission, as illustrated in Figure 3e. The Cu^+^ dopant has been previously explored in the colloidal Cs_2_ZnCl_4_ system to achieve blue emission with a PL peak maximum at 477 nm, which was assigned to Cu^+^ tetrachloride-induced trapped excitons caused by intra-gap states.^41^ The difference in the emission wavelengths of the colloidal nanocrystal systems and the present system is likely caused by the ligand environment of the colloidal system. The present system is completely ligand-free and emits intense violet-blue emission in the solid state. In addition, the FWHM (30 nm) and Stokes shift (74 nm) are relatively small compared to previously reported Cu^+^-containing metal halide systems.^57−60^ Thus, the Cs_3_ZnI_5_:Cu system is highly desirable for solid-state lighting applications because its FWHM is in the range required for International Commission for Illumination.^61^ The shape of the photoluminescence excitation (PLE) spectra acquired at the emission maximum of the Cu-and Sn-doped samples correlates well with the absorption spectra (Figure S9). However, in contrast to the absorption spectra, the peak intensity of the new band that arises from metal doping is higher than the band-edge absorption peak, suggesting that the intra-gap PL originates from the band-edge absorption as well as the absorption of the metal halide formed upon doping.

In the Sn-doped system, the broad emission spectra with a large stokes shift (129 nm) suggest that the PL arises from the STE of [SnI_4_]^2–^ units in the lattice (Figure 3a,b). The broad PL of SnX_4_^2–^ (X = Cl^–^, Br^–^, and I^–^) in organic–inorganic and all-inorganic matrices has been studied, and it was attributed to the intrinsic PL of individual SnX_4_^2–^ species and the structural deformation of regular tetrahedral (C_2V_ symmetry) geometry to the disphenoidal structure (low D_2d_ symmetry) at the excited state that leads to a large stokes shift.^49,62,63^ It is known from the literature that, for the ions with ns^2^ outer electronic configuration (here, it is 5s^2^ for the Sn^2+^ ion), the ground state is ^1^S_0_ and the excited state splits into four energy levels, namely, ^1^P_1_, ^3^P_0_, ^3^P_1_, and ^3^P_2_. The ^1^P_1_ state is a singlet, while the ^3^P_*n*_ (*n* = 0, 1, 2) is a triplet state.^49,63^ According to the spin-selective transition rules, ^1^S_0_ → ^1^P_1_ is an allowed transition, and a ^1^S_0_ → ^3^P_1_ transition is partially allowed due to a spin-orbit coupling for heavy atoms, while ^1^S_0_ → ^3^P_2_ and ^1^S_0_ → ^3^P_0_ transitions are completely forbidden.^49,64^ The ^1^P_1_ → ^1^S_0_ transition emits at a higher energy region, whereas the ^3^P_1_ → ^1^S_0_ transition typically emits at a lower energy region. Therefore, the emission peak of Cs_3_ZnI_5_:Sn at 596 nm is likely caused by the lower energy ^3^P_1_ → ^1^S_0_ transition (Figure 3e).^49,63^ The long PL lifetime further supports the spin-forbidden ^3^P_1_ → ^1^S_0_ transition and thus STE in the Sn-doped system.

### Synthesis and Optical Properties of White Light Emissive CsI:Mn

**Figure 4:**
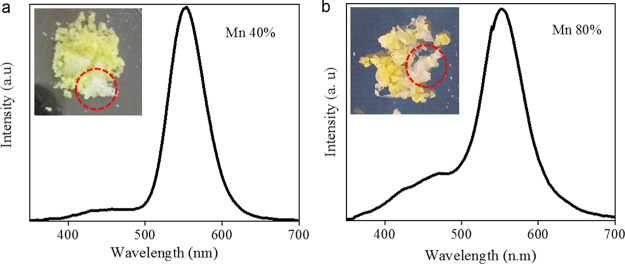
Photoluminescence spectra of Mn-doped Cs_3_ZnI_5_ samples at 40% (a) and 80% (b). Insets are photographs of the crystals prepared. The white color impurity that emits white light under UV light illumination is marked in the photographs.

**Figure 5:**
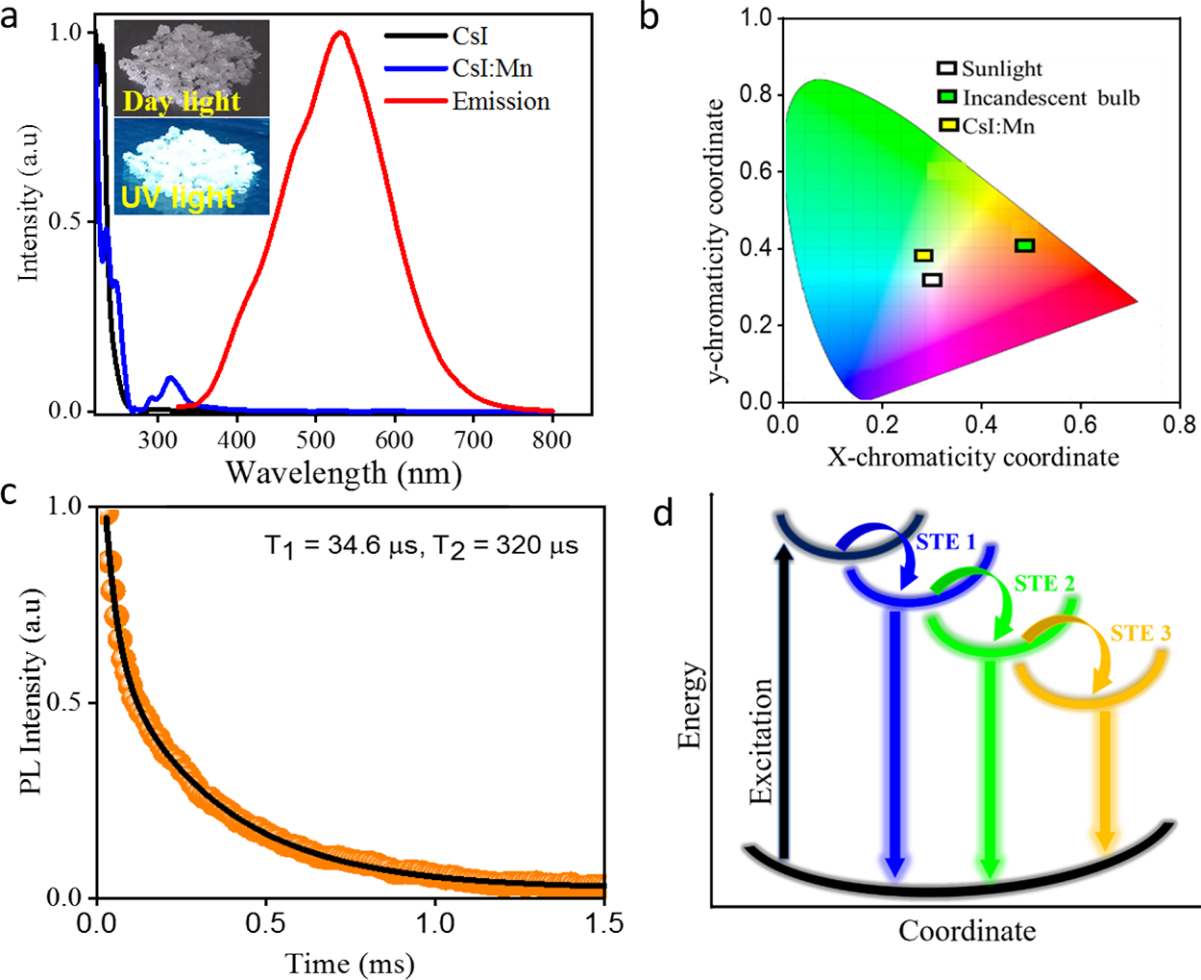
(a) UV–vis absorption and emission spectra of white light emissive Mn-doped CsI sample. The absorption spectrum of the nonemissive pure CsI sample is also provided for comparison. (b) Chromaticity coordinates of the CsI:Mn sample in comparison with sunlight and incandescent bulb. (c) Time-resolved PL lifetime data probed at the emission maximum of the white light. (d) Schematic illustration of the origin of white light emission attributing to the creation of self-trapped emissive states at different intra-gap states upon Mn-doping into CsI.

While studying the effect of Mn-dopant concentration on the emissive properties, surprisingly, we noticed white light emission from a few single crystals (insets of Figure 4a,b). Figure 4 shows the PL spectra of Mn-doped (40 and 80%) Cs_3_ZnI_5_ samples. At high Mn-dopant concentrations, the spectra get broader with a few additional peaks, leading to the white light emission. The new emission between 400 and 500 nm suggests that high Mn-doping leads to the formation of new energy states or crystals with different compositions. We presumed that the broadband emission originates from the formation of Mn-doped CsI impurities. Interestingly, a previous study reported broad emission spectra from Tl-doped CsI samples.^65^ We then synthesized pristine and Mn-doped CsI crystals using CsI and MnI_2_ precursors following the procedure illustrated in Figure 1a. The prepared Mn-doped CsI crystals emit bright white light under UV light illumination. The powder XRD data perfectly match with the Inorganic Crystal Structure Database (ICSD: #98-005-9353) pattern, confirming the successful synthesis of white light emissive Mn-doped CsI single crystals (Figure S12). Subsequently, Tauc plots have shown that the band edge absorption values for CsI and Mn-doped CsI are 5.19 and 3.64 eV, respectively (Figure S13). The pristine CsI sample exhibits broad absorption spectra with clear peaks at 230 and 280 nm (Figure 5a). The Mn-doped CsI shows absorption peaks at 290 and 315 nm in addition to the peak at 230 nm that corresponds to the pristine CsI. The new absorption peaks at lower energies indicate the formation of intra-gap states or new absorption species in the crystal lattice. The corresponding emission spectra were recorded and found that the Mn-doped CsI samples exhibit a broad emission spectrum covering from 350 to 750 nm, while the pristine CsI is nonemissive despite strong absorption of UV light (Figure 5a). Such white light emission spectra have been previously reported for Pb-based low-dimensional organic–inorganic hybrid systems.^35−37^ It is very interesting and exciting to see strong white light emission from a complete inorganic system. It is most likely that the Mn doping in CsI leads to the formation of multiple STE states at different energies, leading to white light emission due to the simultaneous emission of primary colors (Figure 5d).

The PL spectra of the Mn-doped CsI are composed of several small peaks at different wavelengths (Figure S14), indicating STE states of different energies. The time-resolved PL measurements revealed that the charge carrier recombination occurs in tens of microseconds (Figure 5c), which is typical for STE (Figure 5d). The emission spectra are independent of the excitation wavelength, such that the emission is likely to arise from single excitation species (Figure S15). The PL decay fits well with a biexponential curve, and the estimated average lifetime is ∼177.3 μs by integrating two individual lifetimes of 34.6 and 320 μs. Such a long lifetime is likely to occur for broadband white-light emission (Figure 5c).^66,67^ The emission maximum of the white light is ∼540 nm, which resembles the emission of the d-d transitions in tetrahedral Mn(II) halide. However, the PL lifetimes are quite different in both systems (CsI:Mn and Cs_3_ZnI_5_:Mn). Moreover, no sign of tetrahedral Mn(II) is observed in XRD data. Therefore, it is most likely that the peak at 540 nm corresponds to self-trapped states, but we cannot completely omit the contribution of tetrahedral Mn(II) emission. However, in-depth theoretical studies are needed for a better understanding of this all-inorganic white light emissive system. Nevertheless, the Mn-doped CsI crystals have great potential as a solid-state white light source for lightning applications.

The quality of light is often measured with Color Rendering Index (CRI), which defines how natural colors display under an artificial white light source when compared to sunlight. The higher the CRI value, the better the performance of the white light source. Generally, light sources with a CRI of 80 or above are considered good. Here, the Mn-doped CsI crystals exhibit a CRI of 78 (Figure 5b). This valve is far higher than the values of basic fluorescent light sources (∼65) and is comparable to the commercially available LEDs (80).^37,68^ As depicted in the CIE (International Commission on Illumination) 1931 color coordinator diagram, the CsI:Mn system exhibits chromaticity coordinates of (0.30, 0.38) with a correlated color temperature (CCT) of 6530 K that corresponds to cold white light or daylight (Figure 5b). The color coordinates of the CsI:Mn system is very close to the sunlight, which makes it ideal for lightning.

**Figure 6:**
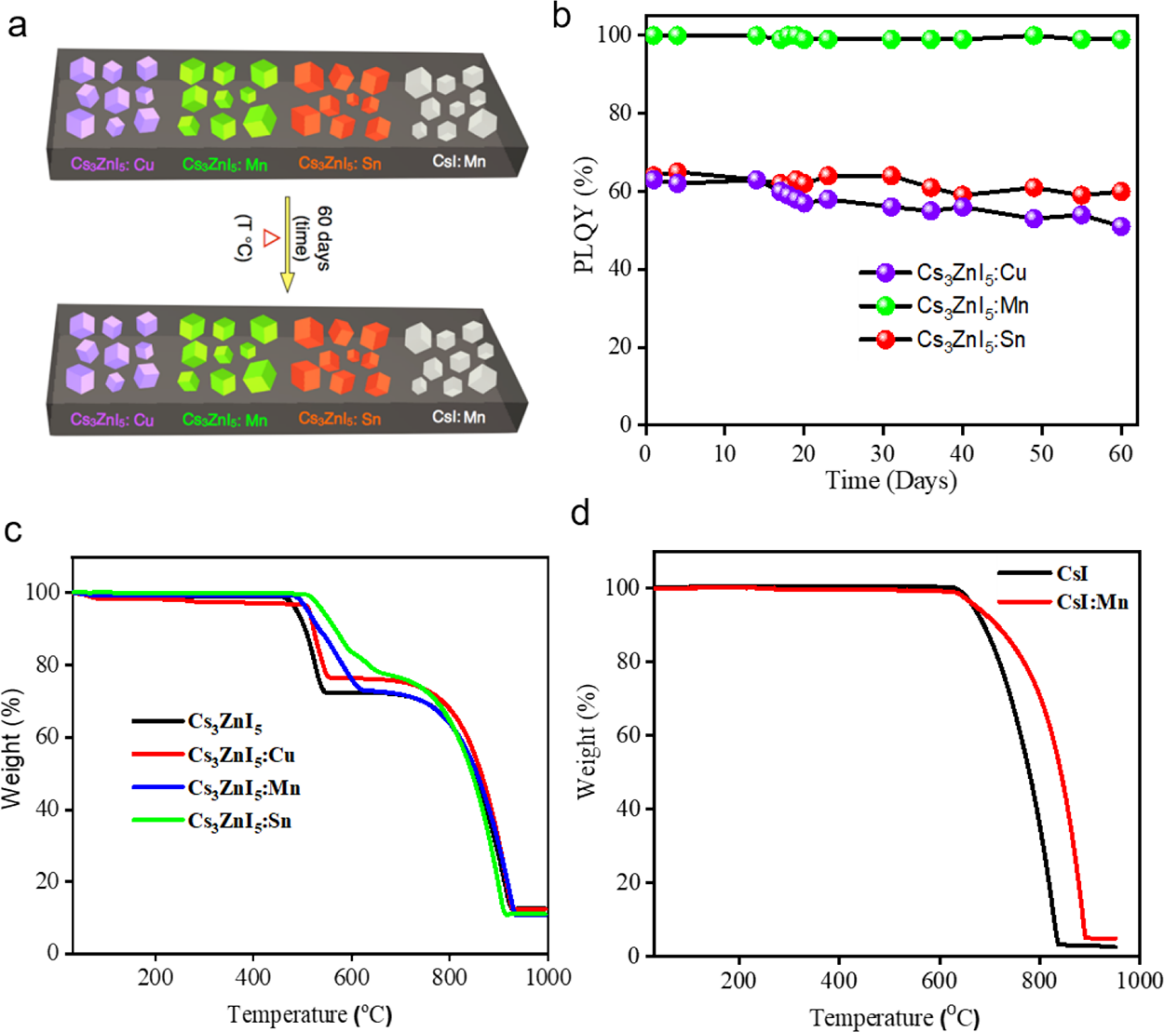
(a) Schematic illustration for the stability studies on doped single crystals with respect to time and temperature. (b) PLQY of Cs_3_ZnI_5_:M (M = Cu, Mn, and Sn) crystals vs time after their preparation. (c, d) Thermogravimetric analyses of Cs_3_ZnI_5_, Cs_3_ZnI_5_:M (M = Cu, Mn, and Sn) and CsI, CsI:Mn to 1000 °C.

For the practical use of light sources, thermal stability, and long-term stability, is important. Therefore, we investigated the thermal stability of the samples studied by thermos gravimetric analysis (TGA) in the range of 30–1000 °C temperature, and the traces are depicted in Figure 6. The CsI:Mn and Cs_3_ZnI_5_:M samples have not shown any significant weight loss up to 630 and 460 °C, respectively (Figure 6c,d). Interestingly, the decomposition temperature slightly increased by 20–50 °C for Cs_3_ZnI_5_:M samples compared to the pristine sample, which is likely due to the structural disorder arising while different ions in the lattice.^46,69^ In addition, differential scanning calorimetry (DSC) measurements were performed to investigate the structural phase transition and find that no significant phase change occurred (Figure S16). Furthermore, the PLQYs and XRD patterns of all the samples remain unchanged after storing them at ambient conditions for 60 days, which demonstrates the excellent stability of the samples (Figure 6a and Figure S17).

## Conclusions

In conclusion, we have reported all-inorganic, Pb-free, doped metal halide-based solid state emitters with extremely high PLQY and tunable emission from violet to green to orange/red and white light emission by varying the dopant or host matrix. The Cu-doping in Cs_3_ZnI_5_ leads to blue emission with 57% PLQY, while the Mn and Sn-doping results in green and orange/red emission with PLQYs 100 and 64%, respectively. Interestingly, the excess Mn-doping results in the formation of white light emissive Mn-doped CsI single crystals. Furthermore, the synthesis of the bright white light emissive crystals results in a pure form with a high color rendering index of 78, which is higher than the basic fluorescent light sources (∼65) and akin to the commercially available LEDs. To the best of our knowledge, this is the first report on the white light emissive all-inorganic metal halide system. The chromaticity diagram also showed coordinated of (0.30, 0.38) with a correlated color temperature (CCT) of 6530 K suitable for “cold” white light for outdoor illumination, and the coordinates are very close to the sunlight. Importantly, the doped-single crystals are highly stable for several months as the PLQY remains unaltered two months after their preparation. Consequently, the results of this work not only demonstrate the tunability of emissive color in 0D metal halides by different dopants, these materials have a tremendous potential to play an important role in forthcoming solid-state lighting and display technologies owing to the Pb-free nature, high PLQY, and extremely high thermal stability.

## Experimental Section

### Materials and Methods

#### Chemicals

Zinc(II) iodide (ZnI_2_, 99.995%) were purchased from the Alfa-Aesar company. Cu(I) iodide (CuI, 99.999%), Mn(II) iodide (MnI_2_, 99.99%), tin(II) iodide (SnI_2_, 99.99%), cesium iodide (CsI, 99.9%), and hypophosphorous acid (H_3_PO_2_, 50% w/w aq. soln.) were purchased from the Sigma-Aldrich company. Hydroiodic acid (HI, 57% w/w aq. soln.) was purchased from TCI chemicals. Ethanol (C_2_H_6_O, 99.9% absolute solution) was purchased from commercial alcohols, Brampton, Canada. All of the chemicals were used as received without any further purification.

### Synthesis of Mn(II)-Doped CsI

The single crystals of Mn-doped CsI were synthesized by following the temperature-lowering procedure.^46^ The CsI_(1–*X*)_MnI_2(*X*)_ (0.259 g, 1 mmol, where *X* = 0.1, 0.2, 0.3, 0.4, 0.5%; here, the weight is based on CsI weight) was dissolved in a mixture of 2 mL of HI and 0.5 mL of H_3_PO_2_ solution in a closed vessel with constant stirring at 150 °C, and after 30 min, the stirring was stopped, and the reaction temperature was lowered from 150 to 27 °C with a rate of 20 °C/h. After 2 h, flake-type transparent single crystals were obtained. The role of H_3_PO_2_ is to prevent the oxidation state of the Mn^2+^ ion. The crystals were separated from the solution and washed with ethanol before use for X-ray diffraction and other spectral studies.

### Synthesis of Cu(I)-, Mn(II)-, and Sn(II)-Doped Cs_3_ZnI_5_

The single crystals of Cs_3_ZnI_5_ and its Cu(I), Mn(II), and Sn(II)-doped samples were synthesized by following the temperature-lowering procedure.^46^ First, ZnI_2_ (0.160 g, 0.5 mmol) was dissolved in 2 mL of HI solution in a closed vessel with constant stirring at 150 °C. To this solution, CsI (0.39 g, 1.5 mmol) was added, and after 30 min, the stirring was stopped, and the reaction temperature was lowered from 150 to 27 °C with a rate of 20 °C/h. After 5 h, needle-type longer-size single crystals were obtained. For the doped Cs_3_Zn_1–*X*_M_*X*_I_5_ (where M = Cu(I) and *X* = 0.5, 1, 1.5, 2, up to 10%; M = Mn(II) and *X* = 1, 2, 3, 4, 5, up to 40%; and M = Sn(II) and *X* = 0.5, 1, 1.5, 2, 2.5%; here, the weight is based on ZnI_2_ weight) single crystals, similar procedure was followed except the use of CuI, MnI_2_, SnI_2_, and H_3_PO_2_ reactants. The amounts of H_3_PO_2_ (0.5 mL) and CsI are constant, while the varied amounts of ZnI_2_, CuI, MnI_2_, and SnI_2_ were used. The role of H_3_PO_2_ is to prevent the oxidation states of Cu^+^, Mn^2+^, and Sn^2+^ ions. The crystals were separated from the solution and washed with ethanol before use for X-ray diffraction and other spectral studies. A similar procedure was also employed to the synthesis of bromide-based Cs_3_ZnBr_5_ crystals by adding CuBr and SnBr_2_ as dopants, and shows nonemissive behavior.

### Characterizations

Single-crystal X-ray data of the samples were collected on an Xtlab Synergy Rigaku oxford diffraction with a HyPix-3000 detector, equipped with graphite monochromoted Mo-Kα radiation (λ = 0.71073 Å), and the X-ray generator was operated at 50 kV at 120 K. Data reduction was performed using CrysAlisPro 171.33.55 software (CrysAlisPro; 2010). The structure was solved and refined using OLEX2^70^/SHELX-2014.^71^ Powder X-ray diffraction data of the samples were collected using a PANalytical model X‘pert3 analyzer using a Cu Kα radiation (λ = 1.540598 Å) source at room temperature, 2 theta range from 5° to 60°. Diffuse reflectance spectroscopy (DRS) studies have been carried out with a Shimadzu UV-2600 UV–visible spectrophotometer. Photoluminescence excitation and emission spectra were carried out using Jasco FP-8500. Time-resolved photoluminescence spectra were performed on the FlouroLog-3, Horiba Jobin Yvon system with xenon lamps as an excitation source. The absolute photoluminescence quantum yields of the samples were measured by using Jasco FP-8500 with an integrating sphere module measurement technique. Thermogravimetric analysis measurements were done with a STA-6000 PerkinElmer instrument. Differential scanning calorimetry (DSC) was carried out with a TA DSC-250 instrument, and the samples were heated at a rate of 10 °C min^–1^. Inductively Coupled Plasma Optical Emission Spectrometer (ICP-OES) is performed on VARIAN 720-ES. Field-Emission Scanning Electron Microscopy (FESEM) images were recorded using a Carl Zeiss Ultra 55 microscope. Energy-dispersive X-ray (EDX) spectra and elemental mappings were recorded using an Oxford Instruments X-Max^N^ SDD (50 mm^2^) system and INCA analysis software. XPS studies were performed in a Thermo Scientific K-Alpha spectrometer equipped with a micro-focused monochromatic X-ray source (Al Kα, spot size ∼400 μm) operating at 70 W. The energy resolution of the spectrometer was set at 0.5 eV at a pass energy of 50 eV, and 5 × 10^–5^ torr base pressure was maintained at the analysis chamber. Low-energy electrons from the flood gun were used for charge compensation.

## Supporting Information Available

Experimental section; single-crystal growth process for CSI:Mn; pristine and Cs3ZnI5:M (where M = Cu, Mn, and Sn); digital images of bulk samples at RT and UV-light; powder X-ray diffraction (PXRD) pattern; EPR studies; XPS spectral analysis; bandgap calculations; UV–visible absorption and emission studies; PXRD, bandgap calculations, and PL studies of CsI:Mn; DSC analysis; stability studies; and crystallographic data (PDF)
