# Supplementary material for: All-Inorganic Lead-Free Doped-Metal Halides for Bright Solid-State Emission from Primary Colors to White Light
Source: ACS Appl Mater Interfaces. 2023 Jul 17;15(29):35206–15. doi: 10.1021/acsami.3c06546 (PMC10375434; doi:10.1021/acsami.3c06546)
Supplement: Supplementary file 1 — am3c06546_si_001.pdf [file am3c06546_si_001.pdf]

Supporting Information

All-inorganic Lead-free Doped-Metal Halides for Bright Solid-state Emission from Primary Colors to White Light

Ramavath Babu,[†]* *Iago López-Fernández,*[‡] *Seelam Prasanthkumar,*[§, #]* *Lakshminarayana Polavarapu*[‡]*

[†]*School of Chemistry, University of Hyderabad, Gachibowli, Hyderabad – 500 046, India*
Email: ramavathbabu10@gmail.com

[§]*Polymer & Functional Materials Division, CSIR-Indian Institute of Chemical Technology (IICT), Tarnaka, Uppal Road, Hyderabad – 500 007, India*
[#]*Academy of Scientific and Industrial Research (AcSIR, Ghaziabad, Uttar Pradesh- 201 002, India.*
Email: prasanth@iict.res.in

[‡]*CINBIO, Materials Chemistry and Physics Group, University of Vigo, Campus Universitario Marcosende, 36310 Vigo, Spain*

Email: lakshmi@uvigo.es

Table of contents

1. Single crystals growth process for CSI:Mn, Pristine and Cs_3ZnI_5:M (where M = Cu, Mn and Sn)
2. Digital images of bulk samples at RT and UV-light
3. Powder x-ray diffraction (PXRD) pattern
4. EPR studies
5. XPS spectral analysis
6. Bandgap calculations
7. UV-visible absorption and emission studies
8. PXRD, Bandgap calculations and PL studies of CsI:Mn
9. DSC analysis
10. Stability studies
11. Crystallographic data

1. **Single crystals growth process for CsI:Mn, Pristine and Cs_3ZnI_5:M (where M = Cu, Mn and Sn):**

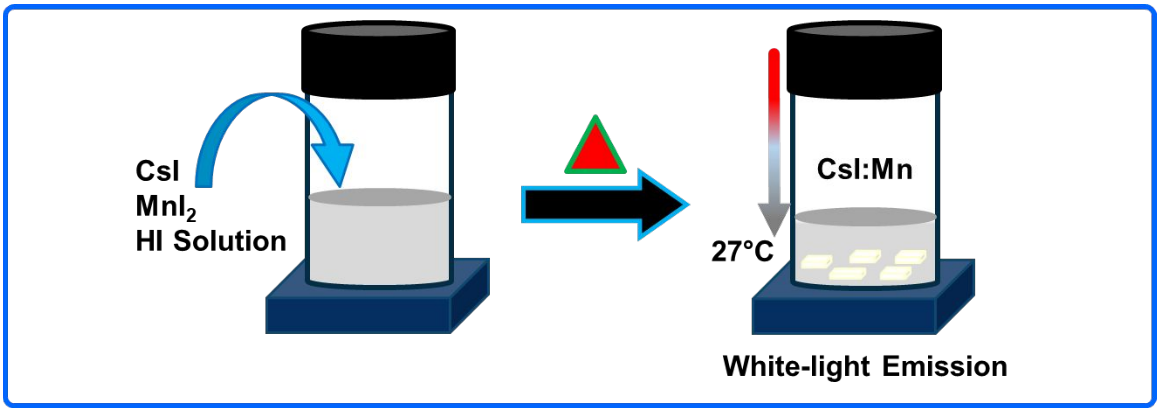

Scheme S1. Schematic illustration for the single crystal growth process of CsI:Mn.

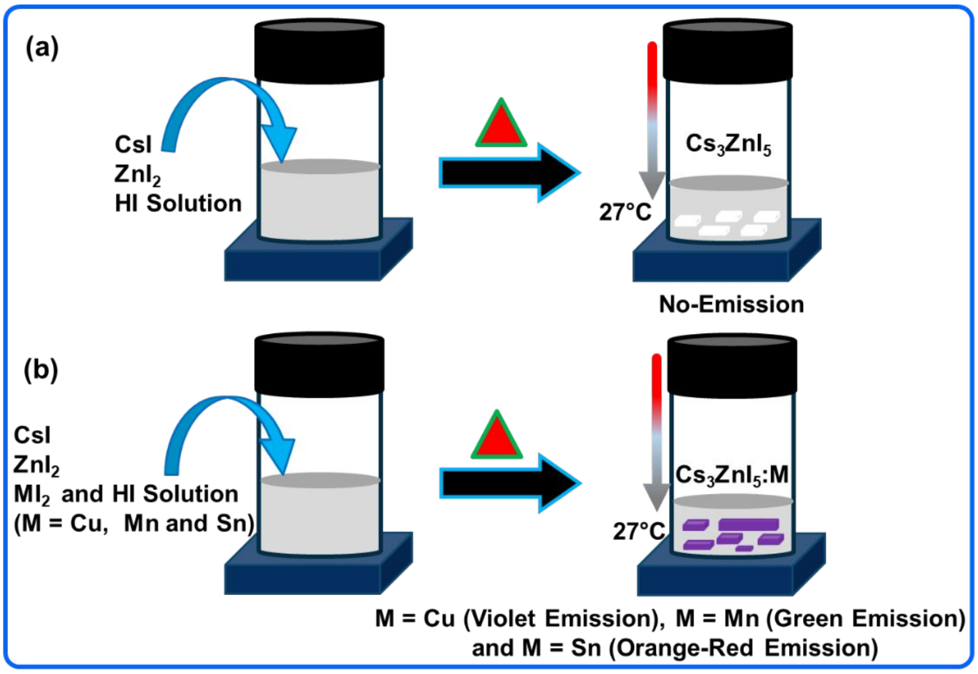

Scheme S2. Schematic illustration for the single crystal growth process, (a) for pristine and (b) for Cs_3ZnI_5:M. (where M = Cu, Mn and Sn).

2. Digital images of bulk samples at RT and UV-light:

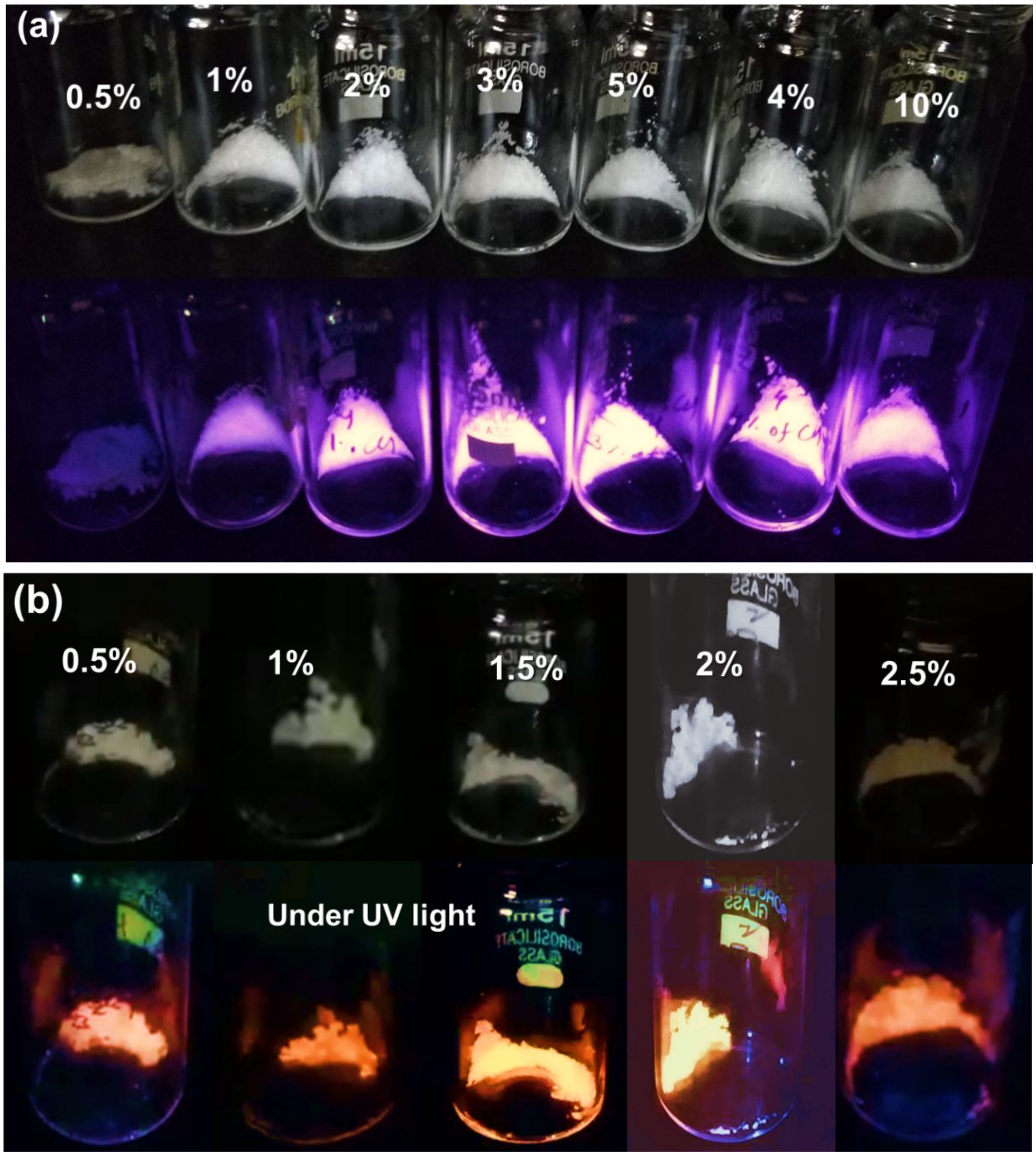

Figure S1. Digital images of bulk samples at RT and UV-light for (a) Cu-doped Cs_3ZnI_5 and (b) Sn-doped Cs_3ZnI_5.

3. Powder x-ray diffraction (PXRD) pattern:

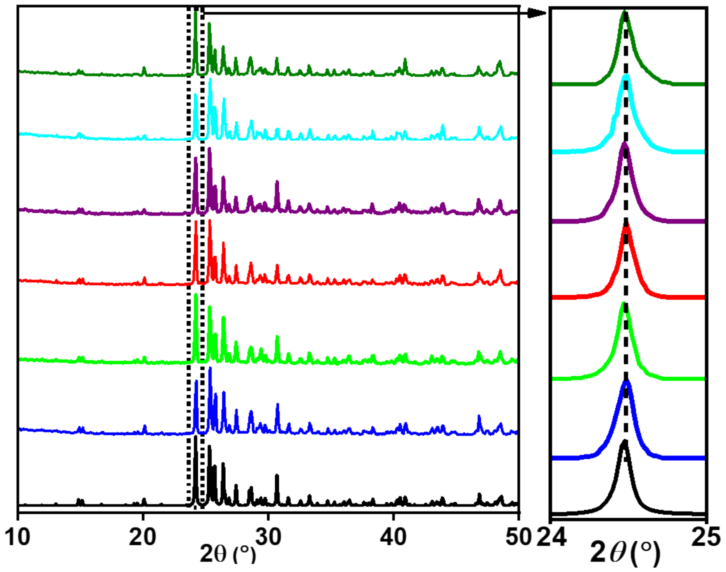

Figure S2. PXRD pattern of Mn-doped Cs_3ZnI_5.

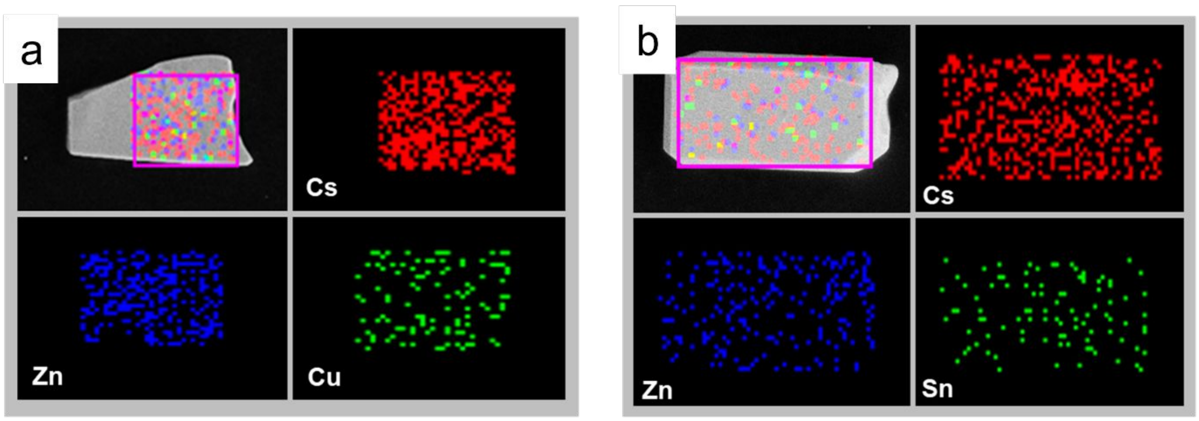

Figure S3. EDX analysis of Cu (a) and Sn-doped (b) Cs_3ZnI_5 samples.

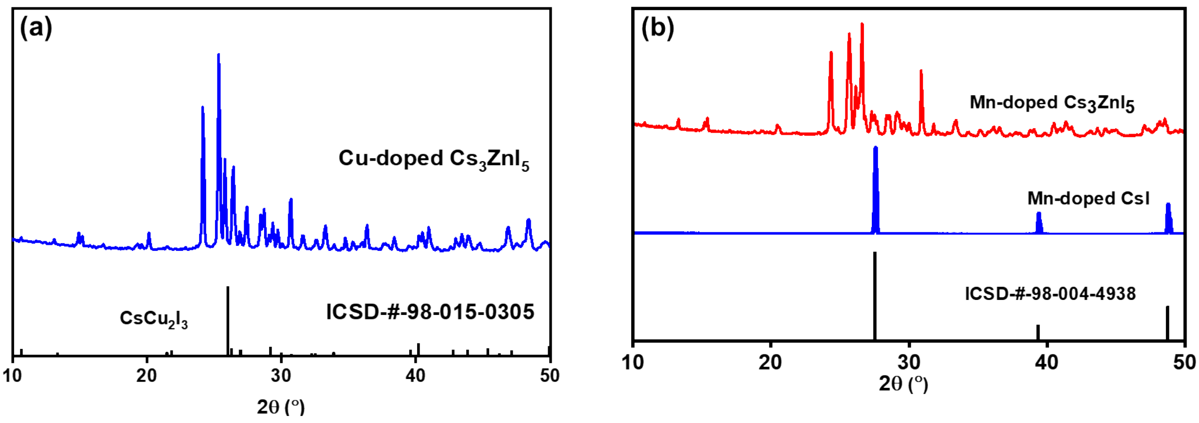

Figure S4. PXRD of (a) Cs_3ZnI_5:Cu and (b) Cs_3ZnI_5:Mn samples at dopant concentrations, the impurity peaks resembles the crystal structures of CsCu2I3 and Mn-doped CsI, respectively

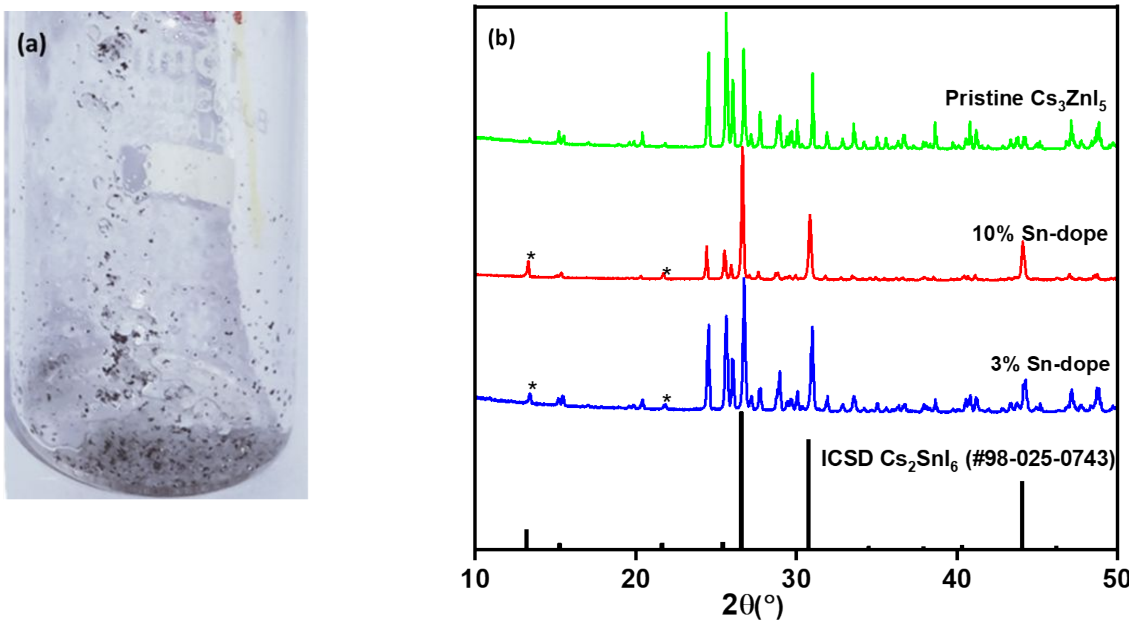

Figure S5. Illustration of impurity (black colour) Cs_2SnI_6 formation in the synthesis of Cs_3ZnI_5:Sn (a) digital image. (b) ICSD data and PXRD pattern for impurity conformation.

4. EPR studies:

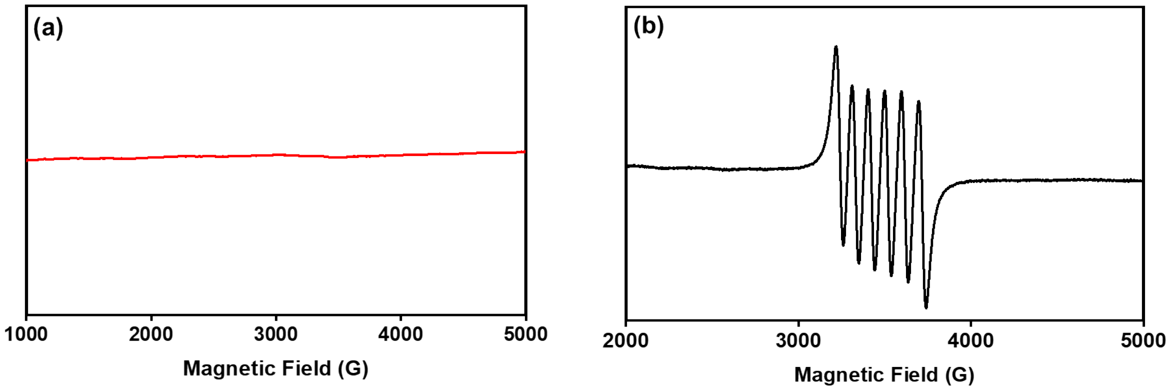

Figure S6. EPR studies for (a) Cs_3ZnI_5:Cu and (b) Cs_3ZnI_5:Mn

5. XPS spectral analysis:

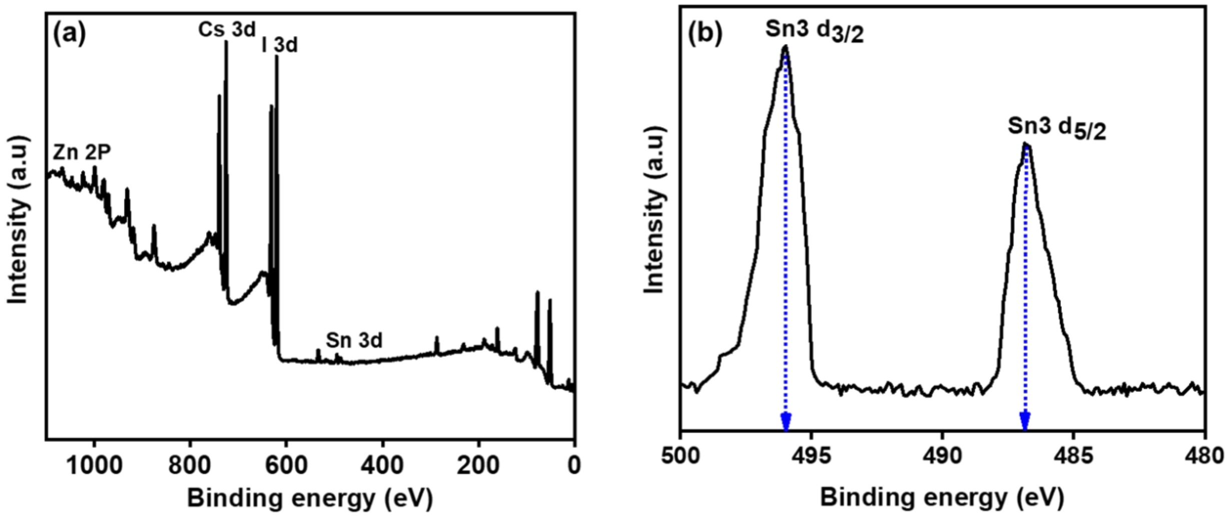

Figure S7. XPS spectral analysis for Cs_3ZnI_5:Sn sample. (a) Overall survey of elements, and (b) is for Sn 3d.

6. Bandgap calculations:

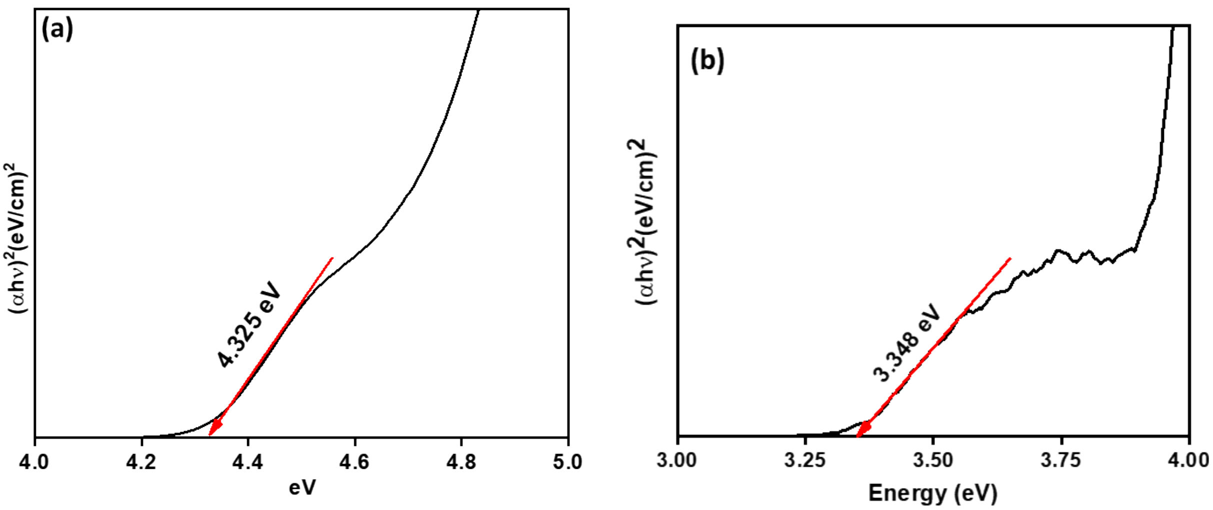

Figure S8. Derived Tauc plots for band gap calculations (a) Pristine and (b) Cs_3ZnI_5:Sn.

7. UV-visible absorption and emission studies:

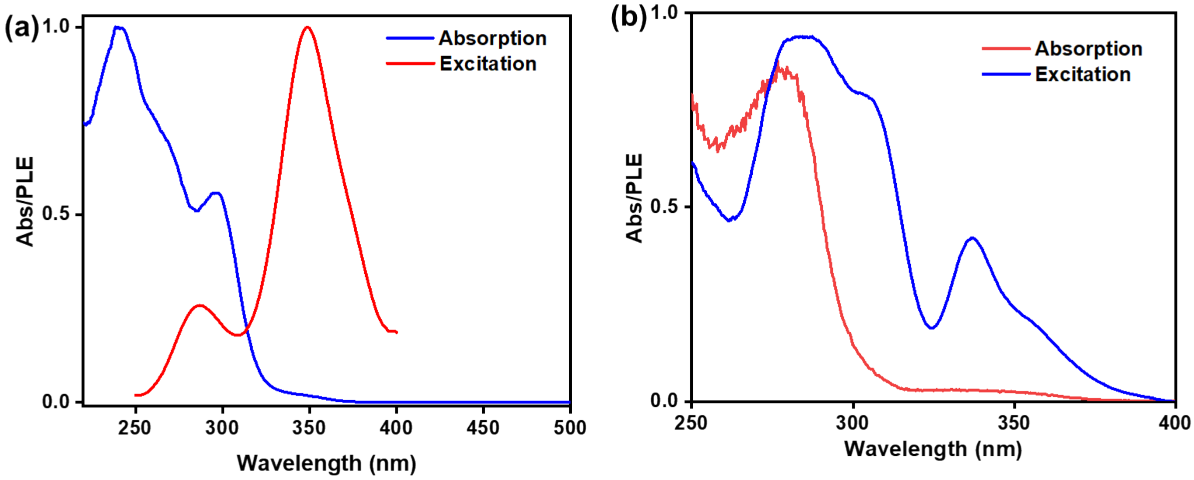

Figure S9. Absorption and PLE spectra (a) for Cs_3ZnI_5:Cu and (b) for Cs_3ZnI_5:Sn at the emission wavelengths of 422 nm and 595 nm, respectively).

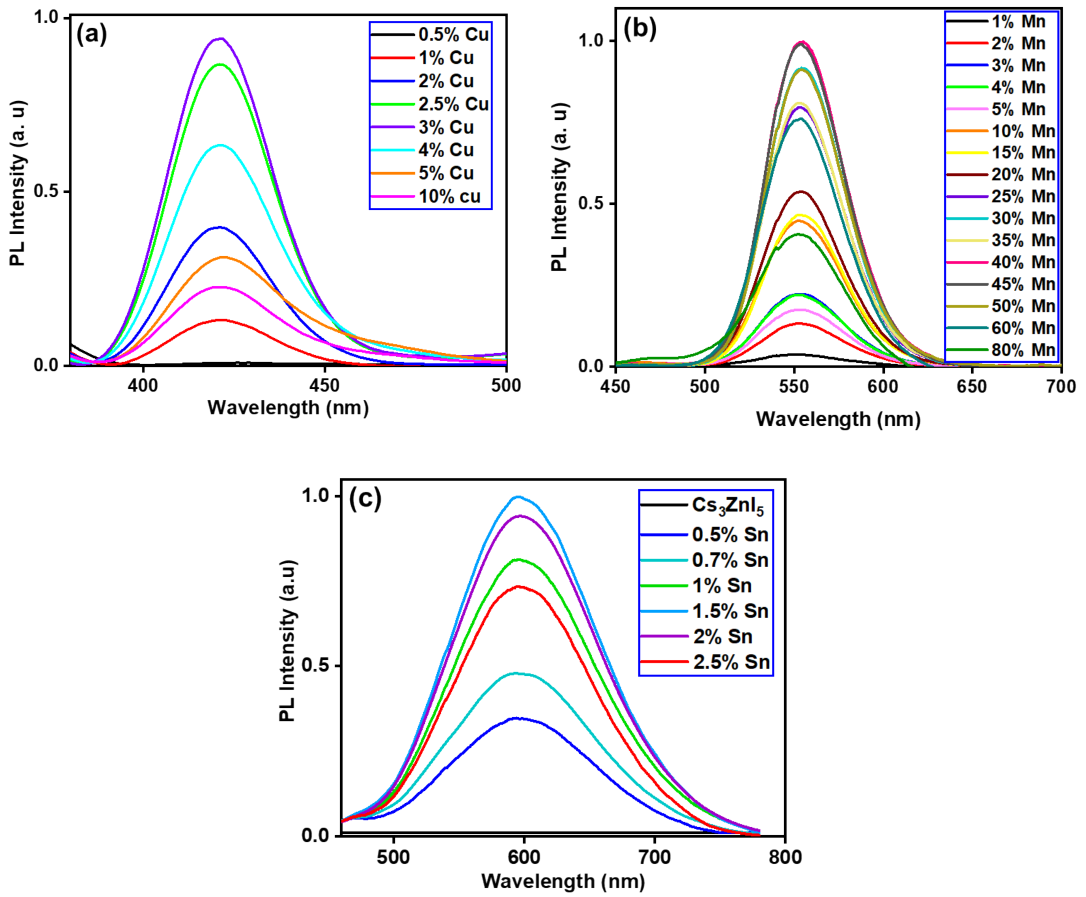

Figure S10. PL spectra at different concentrations (a), (b) and (c) for Cs_3ZnI_5:Cu, Cs_3ZnI_5:Mn and Cs_3ZnI_5:Sn, respectively.

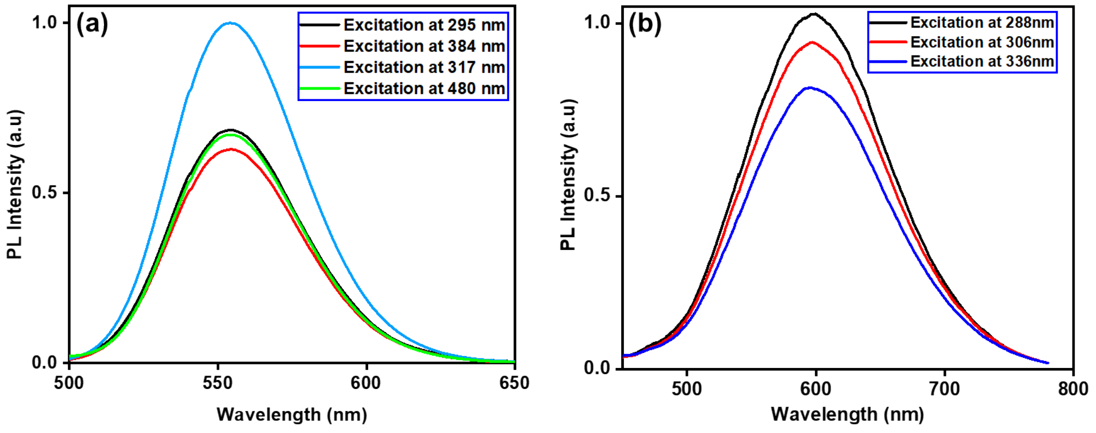

Figure S11. Emission spectra at the different excitation wavelengths (a) for Cs_3ZnI_5:Mn and (b) for Cs_3ZnI_5:Sn.

8. PXRD, Bandgap calculations and PL studies of CsI:Mn

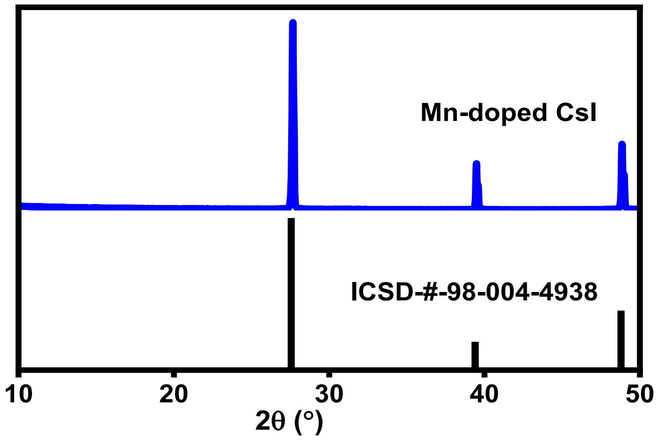

Figure S12. PXRD of Mn-doped CsI fonformation.

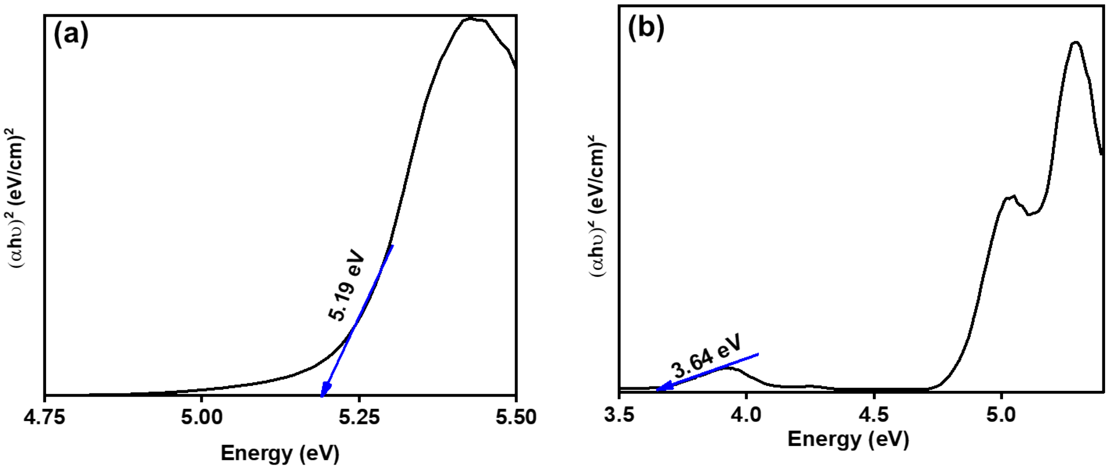

Figure S13. Derived Tauc plots for band gap calculations (a) CsI and (b) CsI:Mn

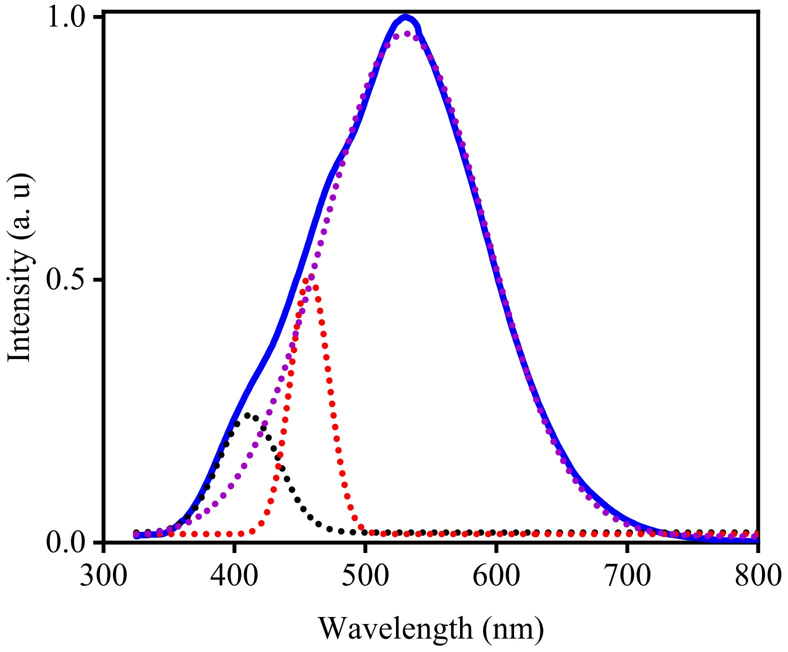

Figure S14. Deconvolution spectra of CsI:Mn.

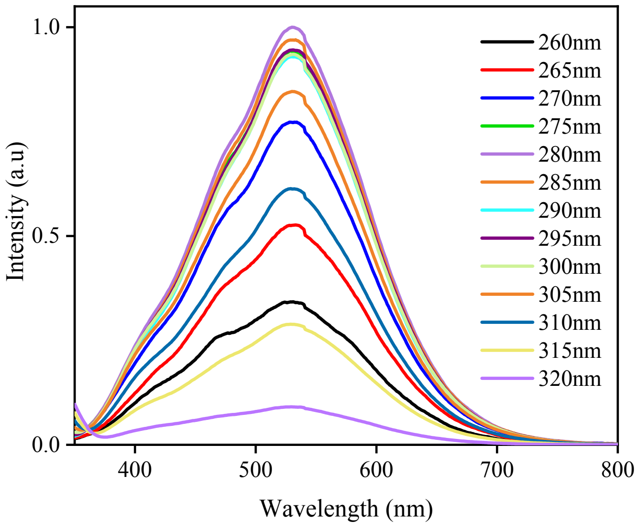

Figure S15. Excitation wavelength-dependent PL spectra of CsI:Mn.The shape of the spectra is independent of the excitation.

9. **DSC analysis:**

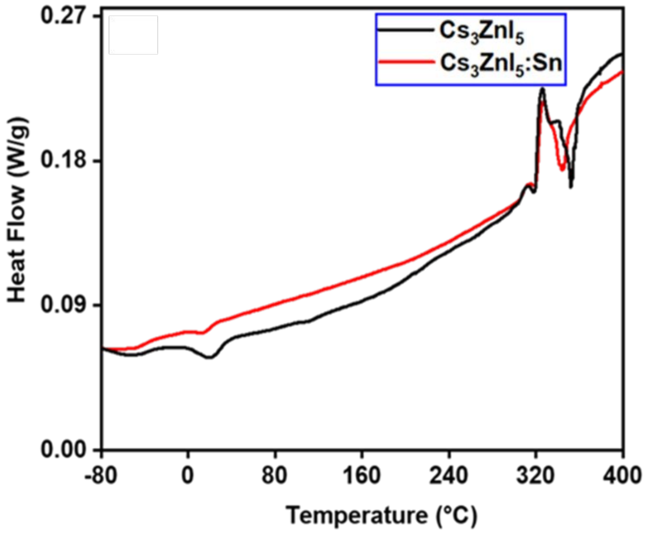

Figure S16. Differential scanning calorimetry (DSC) curves for pristine and Cs_3ZnI_5:Sn.

10. Stability studies based on XRD:

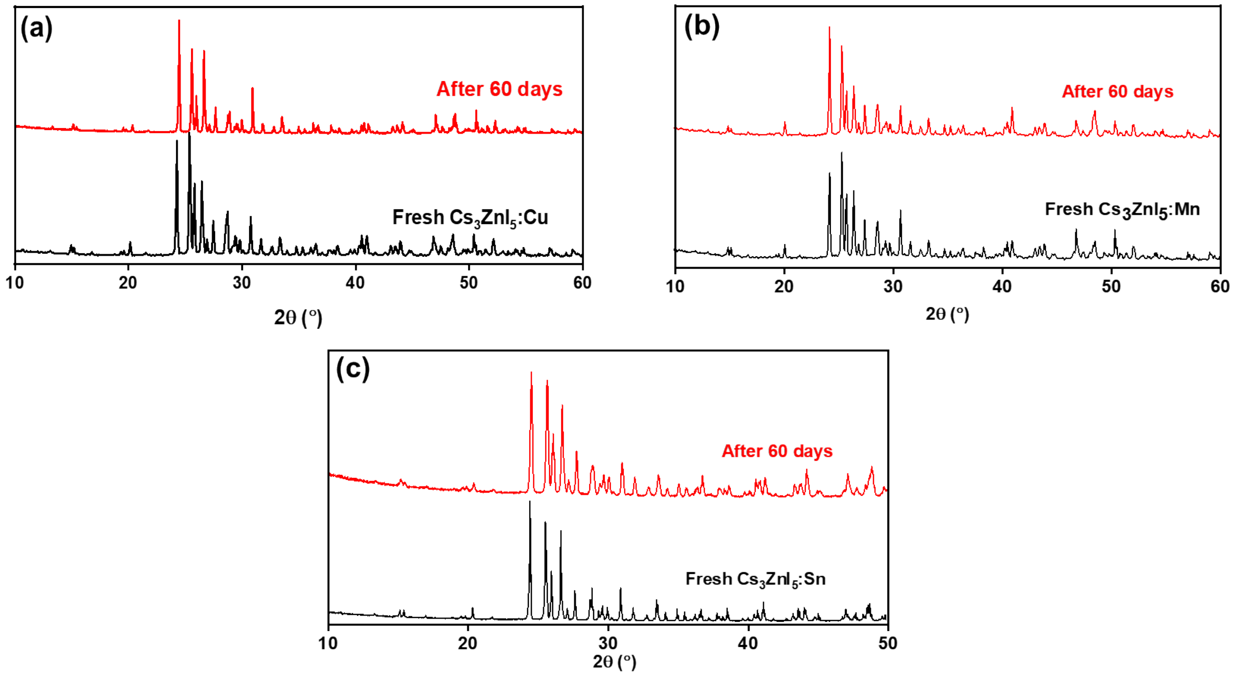

Figure S17. Stability studies for a period of 60 days with PXRD (a) for Cs_3ZnI_5:Cu, (b) for Cs_3ZnI_5:Mn and (c) for Cs_3ZnI_5:Sn.

11. Crystallographic data:

**Table S1.** Crystallographic data and structure refinement parameters for Cs_3ZnI_5, and Cs_3ZnI_5:Sn.		
Formula	**Cs_3ZnI_5**	**Cs_3ZnI_5:Sn**
T [K]	298	298
wavelength [Å]	0.71073	0.71073
crystal system	Orthorhombic	Orthorhombic
space group	*P*nma	*P*nma
a [Å]	9.8556(4)	9.9698(3)
b [Å]	11.6009(5)	11.5646(3)
c [Å]	14.5961(7)	14.6182(4)
α [°]	90	90
β [°]	90	90
γ [°]	90	90
V [$Å^3$]	1668.82(13)	1685.43(8)
Z	4	4
**density [g cm^{-3}]**	4.2993(3)	4.4036(2)
μ [mm^{-1}]	16.838	17.247
F(000)	1840	1840
θ (min, max)	2.2, 26.9	2.3, 27.0
$h_{min, max}$, $k_{min, max}$, $l_{min, max}$	-11: 12 ; -13: 14 ; -18: 18	-12: 10 ; -14: 14 ; -18: 17
no. of ref.	1859	1815
no. of parameters	49	49
no. of unique ref./ obs. ref.	8035/1859	9753/1815
R_{int}	0.180	0.238
R_{all}	0.1200	0.0994
$wR2_{all}$	0.2848	0.3110
G.O.F.	1.17	2.02

**Table S2.** Unit cell parameters for 1.5% Sn-doped Cs_3ZnI_5.			
Formula	**Cs_3ZnI_5:Sn (1)**	**Cs_3ZnI_5:Sn (2)**	**Cs_3ZnI_5:Sn (3)**
crystal system	Orthorhombic	Orthorhombic	Orthorhombic
space group	*P*nma	*P*nma	*P*nma
a [Å]	9.9636(12)	9.9743(11)	9.9634(16)
b [Å]	11.5479(13)	11.5515(14)	11.5576(14)
c [Å]	14.6226(12)	14.6285(17)	14.6226(12)
α [°]	90	90	90
β [°]	90	90	90
γ [°]	90	90	90
V [$Å^3$]	1682.44(5)	1685.45(4)	1683.84(4)
Z	4	4	4

**Table S3.** Unit cell parameters for 2 and 2.5% Sn-doped Cs_3ZnI_5 samples.		
Formula	**2% Sn:Cs_3ZnI_5**	**2.5% Sn:Cs_3ZnI_5**
crystal system	Orthorhombic	Orthorhombic
space group	*P*nma	*P*nma
a [Å]	9.9702(15)	9.9870(9)
b [Å]	11.5422(11)	11.5785(11)
c [Å]	14.6882(13)	14.6861(15)
α [°]	90	90
β [°]	90	90
γ [°]	90	90
V [$Å^3$]	1690.30(5)	1698.19(3)
Z	4	4

**Table S4.** Bond distances (Å) and bond angles (˚) in Cs_3ZnI_5.					
Zn(1)-I(1)	2.592(3)	Zn(1)-I(2)	2.608(4)	Zn(1)-I(3)	2.616(4)
Zn(1)-I(1)(#1)	2.592(3)	I(1)-Zn(1)-I(2)	109.24(9)	I(1)-Zn(1)-I(3)	105.82(9)
I(1)-Zn(1)-I(1)(#1)	115.78(15)	I(2)-Zn(1)-I(3)	110.82(16)	I(2)-Zn(1)-I(1)(#1)	109.24(9)
I(3)-Zn(1)-I(1)(#1)	105.82(9)				

(#1) x, 3/2-y, z

Table S5. Bond distances (Å) and bond angles (˚) in Cs_3ZnI_5:Sn.					
Zn(1)-I(1)	2.596(2)	Zn(1)-I(2)	2.598(3)	Zn(1)-I(3)	2.615(3)
Zn(1)-I(1)(#1)	2.596(2)	I(1)-Zn(1)-I(2)	109.12(8)	I(1)-Zn(1)-I(3)	105.41(8)
I(1)-Zn(1)-I(1) (#1)	116.39(12)	I(2)-Zn(1)-I(3)	111.31(12)	I(2)-Zn(1)-I(1) (#1)	109.12(8)
I(3)-Zn(1)-I(1) (#1)	105.41(8)				
(#1) x, 3/2-y, z					

**Table S6(a). Elemental analysis by ICP-OES for CsI:Mn.**			
S. No	Cs/Mn precursor's ratio (%)	ICP-OES results (% by Mass)	
		Calculated	Experimental
1	100/0	51.15 (Cs)	50.92
2	99.5/0.1	0.051 (Mn)	0.046
3	99/0.2	0.102 (Mn)	0.095
4	98.5/0.3	0.153 (Mn)	0.146
5	98/0.4	0.204 (Mn)	0.195
6	97.5/0.5	0.255 (Mn)	0.234

**Table S6(b). Elemental analysis with ICP-OES and SEM-EDS for Cs_3ZnI_5:Cu**				
S. No	Zn/Cu precursor's ratio (%)	ICP-OES results (% by Mass)		SEM-EDS analysis
		Calculated	Experimental	Composition
1	100/0	5.951 (Zn)	5.921	$Cs_{3.10}ZnI_{5.86}$
2	99.5/0.5	0.035 (Cu)	0.0321	$Cs_{2.78}Zn_{0.92}Cu_{0.03}I_{6.05}$
3	99/1	0.072 (Cu)	0.0571	$Cs_{3.92}ZnCu_{0.06}I_{6.05}$
4	98/2	0.146 (Cu)	0.139	$Cs_{3.13}Zn_{0.12}Cu_{0.14}I_{6.61}$
5	97/3	0.219(Cu)	0.201	$Cs_{3.5}ZnCu_{0.29}I_{6.21}$
6	95/5	0.365 (Cu)	0.323	$Cs_{2.88}Zn_{1.1}Cu_{0.29}I_{5.73}$

**Table S6(c). Elemental analysis with ICP-OES and SEM-EDS for Cs_3ZnI_5:Mn**				
S. No	Zn/Mn precursor's ratio (%)	ICP-OES results (% by Mass)		SEM-EDS analysis
		Calculated	Experimental	Composition
1	100/0	5.951 (Zn)	5.921	$Cs_{3.10}ZnI_{5.86}$
2	99/1	0.072(Mn)	0.068	$Cs_{2.89}Zn_{0.95}Mn_{0.06}I_{6.1}$
3	98/2	0.146(Mn)	0.126	$Cs_{3.12}Zn_{0.99}Mn_{0.10}I_{5.79}$
4	95/5	0.365(Mn)	0.326	$Cs_{3.43}Zn_{0.97}Mn_{0.29}I_{5.31}$
5	90/10	0.731(Mn)	0.711	$Cs_{3.1}ZnMn_{0.76}I_{5.14}$
6	80/20	1.462(Mn)	1.381	$Cs_{2.38}Zn_{1.3}Mn_{1.1}I_{5.22}$
7	70/30	2.193(Mn)	2.019	$Cs_{2.31}Zn_{0.6}Mn_{1.89}I_{5.20}$

8	60/40	2.925(Mn)	2.266	$Cs_{2.10}Zn_{0.62}Mn_{1.98}I_{5.30}$

**Table S6(d). Elemental analysis with ICP-OES and SEM-EDS for Cs_3ZnI_5:Sn**				
S. No	Zn/Sn precursor's ratio (%)	ICP-OES results (% by Mass)		SEM-EDS analysis
		Calculated	Experimental	Composition
1	100/0	5.951 (Zn)	5.921	$Cs_{3.10}ZnI_{5.86}$
2	99.5/0.5	0.029 (Sn)	0.026	$Cs_{2.99}Zn_{0.99}Sn_{0.01}I_{6.05}$
3	99/1	0.059 (Sn)	0.056	$Cs_{3.02}ZnSn_{0.02}I_{5.90}$
4	98.5/1.5	0.089 (Sn)	0.082	$Cs_{3.13}Zn_{0.97}Sn_{0.04}I_{5.85}$
5	98/2	0.119 (Sn)	0.098	$Cs_3ZnSn_{0.05}I_{5.77}$
6	97.5/2.5	0.148 (Sn)	0.110	$Cs_{2.88}Zn_{1.1}Sn_{0.08}I_{5.88}$

Table S7. PLQY values for M(Cu, Mn and Sn)-doped samples and PL lifetimes at their maximum PLQYs

**Cs_3ZnI_5:Cu**				**Cs_3ZnI_5:Mn**				**Cs_3ZnI_5:Sn**			
**Dopant Cu (%)**	**PL-Peak (nm)**	**PLQY (%)**	**Lifetime (µs)**	**Dopant Mn (%)**	**PL-Peak (nm)**	**PLQY (%)**	**Lifetime (µs)**	**Dopant Sn (%)**	**PL-Peak (nm)**	**PLQY (%)**	**Lifetime (ms)**
0.5	422	15		1	555	21		0.5	596	32	
1	"	33		2	"	29		1	"	54	
2	"	48		3	"	42		**1.5**	"	**64**	9.8
**3**	"	**57**	23	4	"	54		2	"	59	
4	"	43		5	"	59		2.5	"	49	
5	"	29		10	"	71					
10	"	20		20	"	84					
				30	"	93					
				**40**	"	**~100**	44				
				50	"	97					
				60	"	74					
				80	"	57					
